# Comprehensive assessment of the disputed *RET* Y791F variant shows no association with medullary thyroid carcinoma susceptibility

**DOI:** 10.1530/ERC-14-0491

**Published:** 2015-02

**Authors:** Rodrigo A Toledo, Roxanne Hatakana, Delmar M Lourenço, Susan C Lindsey, Cleber P Camacho, Marcio Almeida, José V Lima, Tomoko Sekiya, Elena Garralda, Michel S Naslavsky, Guilherme L Yamamoto, Monize Lazar, Osorio Meirelles, Tiago J P Sobreira, Maria Lucia Lebrao, Yeda A O Duarte, John Blangero, Mayana Zatz, Janete M Cerutti, Rui M B Maciel, Sergio P A Toledo

**Affiliations:** 1 Endocrine Genetics Unit (Laboratório de Investigação Médica/LIM-25) of Hospital das Clínicas, University of São Paulo School of Medicine, São Paulo, SP, 05403-010, Brazil; 2 Nursing School, University of São Paulo, São Paulo, SP, Brazil; 3 School of Public Health, University of São Paulo, São Paulo, SP, Brazil; 4 Human Genome Research Center, University of São Paulo, São Paulo, SP, Brazil; 5 Division of Genetics, Genetic Bases of Thyroid Tumors Laboratory, Department of Morphology and Genetics, Federal University of São Paulo, São Paulo, SP, Brazil; 6 Division of Endocrinology, Laboratory of Molecular and Translational Endocrinology, Department of Medicine, Federal University of São Paulo, São Paulo, SP, Brazil; 7 Brazilian National Laboratory of Biosciences, Campinas, SP, Brazil; 8 Centro Integral Oncológico Clara Campal, Hospital Universitário Sanchinarro, Madrid, Spain; 9 Department of Genetics, Texas Biomedical Research Institute, AT&T Genomic Computing Center, San Antonio, Texas, USA; 10 Endocrinology Division, Santa Casa Hospital, São Paulo, SP, Brazil; 11 Laboratory of Epidemiology and Population Sciences, National Institute on Aging, Bethesda, Maryland, USA

**Keywords:** multiple endocrine neoplasias, RET oncogene, MEN2, medullary thyroid carcinoma, genetics

## Abstract

Accurate interpretation of germline mutations of the rearranged during transfection (*RET*) proto-oncogene is vital for the proper recommendation of preventive thyroidectomy in medullary thyroid carcinoma (MTC)-prone carriers. To gain information regarding the most disputed variant of *RET*, ATA-A Y791F, we sequenced blood DNA samples from a cohort of 2904 cancer-free elderly individuals (1261 via Sanger sequencing and 1643 via whole-exome/genome sequencing). We also accessed the exome sequences of an additional 8069 individuals from non-cancer-related laboratories and public databanks as well as genetic results from the Catalogue of Somatic Mutations in Cancer (COSMIC) project. The mean allelic frequency observed in the controls was 0.0031, with higher occurrences in Central European populations (0.006/0.008). The prevalence of *RET* Y791F in the control databases was extremely high compared with the 40 known *RET* pathogenic mutations (*P*=0.00003), while no somatic occurrence has been reported in tumours. In this study, we report new, unrelated Brazilian individuals with germline *RET* Y791F-only: two tumour-free elderly controls; two individuals with sporadic MTC whose Y791F-carrying relatives did not show any evidence of tumours; and a 74-year-old phaeochromocytoma patient without MTC. Furthermore, we showed that the co-occurrence of Y791F with the strong *RET* C634Y mutation explains the aggressive MTC phenotypes observed in a large affected family that was initially reported as Y791F-only. Our literature review revealed that limited analyses have led to the misclassification of *RET* Y791F as a probable pathogenic variant and, consequently, to the occurrence of unnecessary thyroidectomies. The current study will have a substantial clinical influence, as it reveals, in a comprehensive manner, that *RET* Y791F only shows no association with MTC susceptibility.

## Introduction

MEN2 (MIM #164761) is a dominantly inherited multiglandular tumour syndrome that presents with a high penetrance of medullary thyroid carcinoma (MTC; observed in virtually 100% of cases), phaeochromocytoma (50%) and parathyroid adenoma and/or hyperplasia (20%) (Eng *et al*. 1996). Germline gain-of-function mutations of the rearranged during transfection (*RET*) proto-oncogene, mostly occurring in exons 8, 10, 11, 13, 14, 15 and 16, activate RET via autophosphorylation of the transmembrane receptor tyrosine kinase; these mutant receptors are highly expressed in MEN2-related tissues, leading to tumour growth. The International Consortium on MTC of 1996 (Eng *et al*. 1996), the 2001 Consensus on MENs (Brandi *et al*. 2001) and the updated American Thyroid Association (ATA) Consensus on MTC (Kloos *et al*. 2009) recommend that individuals carrying a germline disease-causing *RET* mutation should be subjected to early prophylactic total thyroidectomy, which is currently the only effective approach for preventing the progression of MTC. For the purpose of molecular diagnosis and for adequate surgical management of patients and their relatives, it is crucial to know whether a *RET* gene variant identified via genetic testing is a benign polymorphism, a variant of unknown clinical significance or a pathological disease-causing mutation (Toledo *et al*. 2006).

Biased genetic analyses of highly selected patients, in addition to relatively small numbers of controls, may result in a misunderstanding and misclassification of the pathogenicity of rare variants of medically actionable genes (Weber & Eng 2005). In this context, there is currently a great debate in the literature regarding the potential pathogenicity of the c.2372A>T Y791F variant in exon 13 of *RET* (Berndt *et al*. 1998, Fitze *et al*. 2002, 2004, Gimm *et al*. 2002, Brauer *et al*. 2004, Plaza-Menacho *et al*. 2007, Tamanaha *et al*. 2007, Vestergaard *et al*. 2007, Eng 2010, Erlic *et al*. 2010, Cerutti & Maciel 2013, Pęczkowska *et al*. 2013, Rich *et al*. 2014).

Recently, we described nine MEN2A families carrying both the classical *RET* C634Y mutation and the *RET* Y791F variant, which revealed a previously unrecognised role for Y791F in the MEN2 syndrome. Although further analysis is still needed, our results indicate that Y791F may modify the effects of other concurrent *RET* mutations and may therefore act as a modifier variant (Toledo *et al*. 2010). However, the role of RET Y791F-only in MTC/MEN2-related pathogenesis and susceptibility is currently a matter of dispute (Vestergaard *et al*. 2007, Kloos *et al*. 2009, Eng 2010, Erlic *et al*. 2010, Rich *et al*. 2014 and Supplementary References, see section on supplementary data given at the end of this article).

The current study represents the largest analysis focusing on the *RET* (ATA-A) Y791F variant performed to date, including new data from Sanger and next-generation sequencing analyses of 2904 healthy adult/elderly individuals. We also accessed genetic and genomic findings from multiple public databases, resulting in the analysis of a total of 11 544 healthy individuals and 18 163 cancer samples.

## Subjects

Written informed consent was obtained from the subjects, in accordance with the protocols approved by the Institutional Review Board of each participating centre.

### Cohorts of new investigated controls

In this study, we report data from 2904 previously uninvestigated individuals from three tumour-free adult/elderly cohorts. The first cohort comprised 1261 healthy adult/elderly Brazilians (54% females and 46% males), with a mean age of 65.2 years. Blood DNA samples from these individuals were subjected to Sanger sequencing of *RET* exon 13 in our laboratory at the University of Sao Paulo School of Medicine. This cohort included samples from the School of Public Health of the University of Sao Paulo and control samples from the Experimental Oncology Laboratory (LIM-24) of the University of Sao Paulo School of Medicine. The ethnicity of the tumour-free adult/elderly subjects was 79% White/White Latino and 10% African and mixed Black/Caucasian.

The second cohort has been designated the SABE60+ cohort and is a control group that was assembled and subjected to whole-exome sequencing by the Human Genome and Stem Cell Research Center (HUG-CELL) of the University of Sao Paulo. SABE60+ consists of 604 Brazilian individuals from Sao Paulo City (214 males and 390 females), with minimum and maximum ages of 63.3 and 92.2 respectively, and an average age of 74.2±6.9 years. The majority of the individuals in this cohort were White (57.62%), followed by mixed Black/Caucasian (18.38%), Black (7.45%), Asian (1.99%) and Amerindian (0.33%). Approximately 7% (6.62%) of the individuals answered positively to belonging to another ethnicity, and 7.62% did not answer this question.

The third cohort investigated in this study is the T2D-GENES cohort, which comes from a large collaborative study composed of 1039 completely sequenced individuals. The primary focus of this study group is the discovery of new genes associated with the development of type 2 diabetes using the information provided by large human pedigrees. Each sequenced pedigree contains between 22 and 86 individuals distributed across three to five generations. The sample population is composed of Mexican–American (48.9% males) individuals living in San Antonio, Texas (SATX), who are part of the large San Antonio Family Study (SAFS) project, which has been ongoing for more than 25 years, with a primary focus on cardiovascular diseases.

### Public databases

Genetic and genomic data from the following non-tumour-enriched public databases were accessed: i) the NHLBI GO Exome Sequencing Project; ii) the database of single nucleotide polymorphisms (dbSNP) of the National Center for Biotechnology Information (NCBI), National Library of Medicine and iii) Ensembl (European Bioinformatics Institute, EBI, and European Molecular Biology Laboratory, EMBL), from the Wellcome Trust Sanger Institute (WTSI). The genotyping results for the 1000 European–American controls reported by Erlic *et al*. (2010) were also included.

In addition, genetic and genomic findings from the cancer-specific Catalogue of Somatic Mutations in Cancer (COSMIC) database were also accessed, while variant classifications were obtained from the databases of the ARUP MEN2/*RET* program of the Department of Pathology, University of Utah (Margraf *et al*. 2009).

All of the data from the databases were obtained between 20 July and 23 July 2014, and the websites for each database are listed at the end of the article.

### Literature review

The PubMed and Google Scholar databases were used for the literature review. Space limitations prevented us from providing more thorough coverage of the topic, and we apologise to those whose work could not be cited. An extended list of the studies on *RET* Y791F is included as online supplementary data.

## Methods

### DNA extraction, PCR and Sanger sequencing

Blood DNA was extracted using the salting out method and a Qiagen kit (Qiagen). PCR amplification of *RET* exon 13 was carried out using 10 ng of gDNA and the following primers: F: 5′-AACTTGGGCAAGGCGATGCA-3′ and R: 5′-AGAACAGGGCTGTATGGAGC-3′. The PCR products were run on a 1% agarose gel, and specific 277 bp amplicons (Supplementary Fig. 3, see section on supplementary data given at the end of this article) were then purified using the ExoSap-IT enzyme (USB Co., Cleveland, OH, USA). BigDye Terminator v.3.1 cycle sequencing (Applied Biosystems) was employed to sequence the reaction products, and the samples were run on an ABI Prism 3130xl – Genetic Analyzer (Applied Biosystems).

### Whole-exome and whole-genome sequencing

Whole-exome sequence data were generated using Agilent SureSelect v2 technology for targeted exon capture, and the obtained reads were aligned using the Burrows-Wheeler Aligner (BWH) tool. Piccard was employed to convert, sort and index the aligned data files; the sequence quality scores were recalibrated using Genome Analysis Toolkit (GATK); and annotation of the variants was performed using Annovar.

Whole-genome sequencing (WGS) of 586 individuals was performed externally by Complete Genomics, Inc. (CGI, Mountain View, CA, USA) via a sequence-by-ligation method. The paired-end sequencing reads (70 bp) were mapped using the human reference genome (V 37.2), with a mean coverage of 60×. Variant calling was performed by CGI using version 2.0.3.1 of their proprietary pipeline. The false discovery rate estimates for SNP calls on the CGI platform ranged from 0.2 to 0.6%. The variant calls within the WGS were processed using CGA Tools software (version 1.5.0, build 31), made available by CGI. The WGS information was employed together with the pedigree information and a previously generated genome-wide association chip for effective imputation of offspring using the MACH Bayesian imputation algorithm (Li 2010). The final sample comprised 1039 individuals genotyped using nearly 23 million SNPs.

### Protein structure analysis

The structure of the RET receptor was generated based on the crystal structure of the RET tyrosine kinase domain bound to adenosine that has been recently deposited in the RCSB-PDB protein databank (ID: 4CKJ, Plaza-Menacho *et al*. 2014) and using the software program YASARA (Krieger *et al*. 2009). The RET domains were determined using the Conserved Domain Database (CDD), and a figure was generated with DOG 2.0 (Ren *et al*. 2009).

### *In silico* pathogenic prediction

Four packages with different algorithms were used to investigate the pathogenicity of RET Y791F *in silico*: SIFT; PMUT; Align-GVGD; and POLYPHEN2 (the websites are listed at the end of the article).

### Statistical analysis

Statistical analysis was performed by O Meirelles using IBM SPSS (www-01.ibm.com/software/analytics/spss/).

## Results

### *RET* Y791F in the tumour-free cohorts

**Figure 1:**
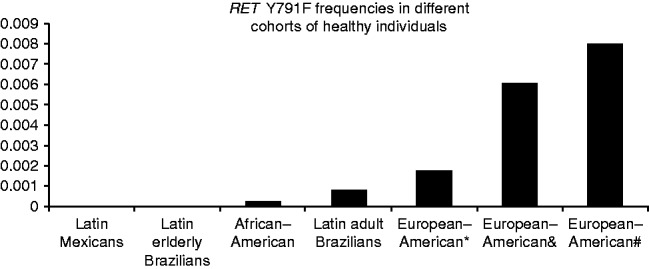
*RET* Y791F frequencies in different control populations/cohorts. The highest frequencies are observed in the populations from central Europe, especially Germany. Details of the populations are provided in Supplementary Table 1.

The analyses of 22 138 alleles from the control subjects revealed that Y791F is a very rare SNP, with a frequency that varies greatly among the analysed populations (Fig. 1 and Supplementary Table 1, see section on supplementary data given at the end of this article). In accordance with an initial report from Europe (Berndt *et al*. 1998), the three European–American cohorts of control individuals presented the highest frequencies, which ranged from 0.0017 to 0.008. The three Latin cohorts together with the African–American population presented the lowest frequencies of *RET* Y791F, ranging from 0 to 0.0007 (Supplementary Table 1). Notably, both the original Y791F MTC cases reported by Berndt *et al*. (1998) and the screen of 1000 controls conducted by Erlic *et al*. (2010), which identified the highest allelic frequency among the controls, are from Germany. In addition, as discussed later in this study, the largest cohort of patients carrying the Y791F variant also comes from Germany, indicating a probably higher prevalence of the Y791F variant in this country. Interestingly, one of the healthy Y791F-only Brazilian individuals reported in this study and the two unrelated Brazilian cases with sporadic MTC who also carried the variant (descriptions of these individuals are provided later in this report) were direct descendants of Germans. In addition, at least two out of the nine Brazilian MEN2A families with C634Y/Y791F that we had previously reported were of German descent (Toledo *et al*. 2010, Valente *et al*. 2013). However, no cases harbouring C634Y/Y791F have been reported outside of Brazil thus far.

**Figure 2:**
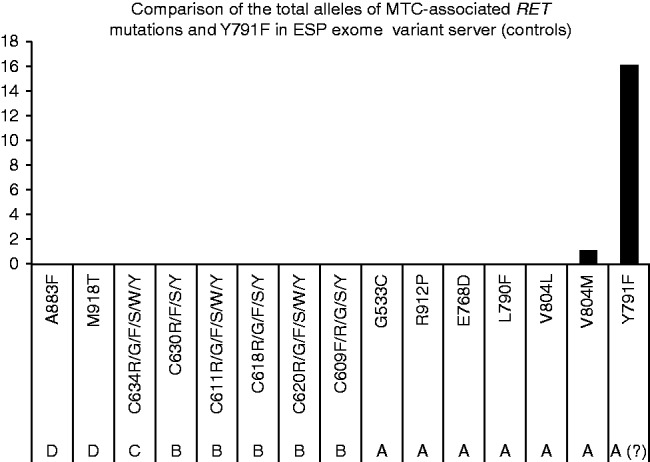
No *RET* ATA-B, C or D alleles were found in the ESP control databases. Only one instance of the V804M allele (ATA-A) was found among the 13 000 sequenced alleles, indicating a very low frequency of *bona fide* MTC-related *RET* mutations in healthy individuals. In contrast, 15 alleles of Y791F were found within the ESP control databases, indicating that the frequencies of this specific variant behave differently, possibly as an accompanying genetic modifier or as a benign polymorphism.

Owing to possible incomplete assessment of the phenotypic features of affected individuals or late disease onset, the control databases may conceivably include patients with rare diseases such as (familial) MTC and MEN2 syndrome who carry pathogenic *RET* variants. To explore the chance of this situation occurring, we assessed whether the Exome Sequencing Project (ESP) exome variation database (which is not a tumour-enriched databank) included any classical disease-associated *RET* mutations. No alleles corresponding to strong *RET* mutations, classified as an MTC level-3 risk by the consensus on MENs or as levels C-D by the ATA MTC guidelines, were found in the ESP control databases (Fig. 2 and Supplementary Fig. 1, see section on supplementary data given at the end of this article). Notably, one allele of the low-risk V804M mutation, which is classified as MTC risk 1/ATA-A, was found among the 13 000 *RET* alleles from the ESP database. Valine 804 is a gatekeeper residue that is critical for ATP binding and activation of the RET kinase receptor. Protein conformation analyses have revealed that the V804M and V804L mutations modify the ATP-binding pockets of RET, facilitating ATP binding and conferring resistance to the multikinase inhibitor vandetanib, which is approved for the treatment of advanced MTC (Carlomagno *et al*. 2004, Wells *et al*. 2010). The identification of heterozygous *RET* V804M in the controls may be due to the relatively mild phenotype or late onset of MTC that is frequently associated with this variant (Feldman *et al*. 2000). The ESP dataset also included one allele of each of the three *RET* variants G321K, K666E, and R866W. These three last variants are very rare and are noted on the ATA guidelines as ‘mutations based on limited families/case reports and may represent variants of unknown significance’ (Kloos *et al*. 2009).

In contrast to the absence/very low frequency of *bona fide RET*-activating mutations in the ESP dataset, the Y791F variant was present in 14 individuals, one of whom was homozygous (N alleles=15). This allelic frequency is significantly higher than that observed among the remaining *RET* mutations (*P*=0.00003).

### Previously unreported tumour-free elderly individuals harbouring *RET* Y791F

In this study, we report two Brazilian *RET* Y791F carriers who were identified during the genetic screening of the healthy elderly controls by Sanger sequencing performed in our laboratory. The first subject is a 76-year-old woman who has never developed tumours and presents no health complications. She has been followed by the Hospital das Clínicas in Sao Paulo (HC-FMUSP) for routine check-ups. Her calcitonin level was undetectable (<2 pg/ml) in September 2014, and she reports no history of tumours in her family. Her father was German and died at the age of 75 due to a heart attack. Her 45-year-old daughter (an only child) was invited to come to the hospital to provide blood for biochemical examinations and genetic testing; her calcitonin level was <2 pg/ml, and sequencing analysis revealed that she harboured the WT allele.

Less information is available regarding the second subject with *RET* Y791F. She was also followed by HC-FMUSP for routine check-ups, and her medical report shows no tumour development or presence of other serious clinical features. She died in 2008 at the age of 94.

In accordance with our data on elderly individuals carrying *RET* Y791F without any history of tumours, a recent study that analysed the exome data of 44 centenarian Ashkenazi Jews has found two individuals (2/44=0.045) harbouring the *RET* Y791F variant (Freudenberg-Hua *et al*. 2014).

### Evaluation of *RET* Y791F in MTC, MEN2, hyperparathyroidism (HPT) and phaeochromocytoma shows no association with susceptibility to endocrine neoplasia

Berndt *et al*. (1998) first reported the presence of the Y791F variant in MTC patients in 1998. In addition to the mutational hotspots in exons 10 and 11, the authors reported exon 13 as a new potential hotspot following the identification of the L790F and Y791F variants in patients with familial MTC. In the original paper, the two *RET* Y791F families were small and included a combined total of only three clinically affected individuals (Berndt *et al*. 1998). The association linking Y791F to familial MTC/MEN2 is based on limited and selected cohorts. More importantly, there is no convincing data in the literature showing co-segregation of Y791F with MTC (Supplementary References).

Additionally, Gimm *et al*. (2002) analysed German patients with mutations in codons 790 and 791 and suggested a mild form of the disease.

Through a review of the literature, we found two large families reported to carry *RET* Y791F; one family is from Denmark with Central Europe descendants, and the other is from Brazil. Vestergaard *et al*. (2007) initially identified a Danish young case with goitre carrying *RET* Y791F and subsequently performed genetic screening of a total of 27 family members. It is noteworthy that 15 of the family members were Y791F carriers, and none exhibited abnormal clinical or biochemical results. The calcitonin levels of the carriers and non-carriers in this family did not differ significantly, and total thyroidectomy was postponed (Vestergaard *et al*. 2007). The second kindred were a large Brazilian *RET* Y791F family with 17 carriers, ten of whom were diagnosed with MTC, including cases with the early and aggressive phenotype (Tamanaha *et al*. 2007). Although this family was initially described as an Y791F-only family, a subsequent analysis indicated that these patients also harboured the *bona fide* activating germline *RET* C634Y mutation (Cerutti & Maciel 2013, Valente *et al*. 2013). These results are in accordance with the previous report of C634Y-Y791F *cis* being found in four Brazilian MEN2A families (Toledo *et al*. 2010).

The largest reported cohort of patients carrying *RET* Y791F was described by Frank-Raue and colleagues and comprised 22 German MTC/MEN2 cases and 34 screened relatives. The authors concluded that Y791F carriers develop milder clinical features and achieve higher cure rates compared with carriers of the codon 790 and 804 mutations (Frank-Raue *et al*. 2008). No control samples from the German population were analysed in this study. Patients with Y791F who display hyperparathyroidism and no MTC have been reported as well (Vierhapper *et al*. 2005). Another study identified *RET* Y791F in German patients with glioblastoma multiforme and gastric and pancreatic cancers who showed no clinical features of MTC or MEN2 (Rückert *et al*. 2011).

In accordance with the results of studies by Erlic *et al*. (2010) and Pęczkowska *et al*. (2013) showing occasional phaeochromocytoma cases with co-occurrence of the *RET* Y791F variant and pathogenic variants in other phaeochromocytoma-related genes, we identified a 68-year-old male with phaeochromocytoma carrying the *RET* Y791F variant and the new germline variant D236N in the *SDHB* gene. Nevertheless, the pathogenicity of the latter variant is still uncertain.

### Two new MTC patients harbouring *RET* Y791F without a family history of endocrine neoplasias

In this study, we report a 37-year-old female followed by the MEN clinic of the Federal University of Sao Paulo (UNIFESP) who presented a thyroid nodule with cytological results indicative of Hürthle cell neoplasm and an extremely high calcitonin level (2079 pg/ml, normal=8.5/5.0 pg/ml). During physical examination, the patient showed no cutaneous lichen amyloidosis, marfanoid habitus or mucosal neuromas. She underwent total thyroidectomy with prophylactic central neck dissection. A pathological analysis revealed a 2.5 cm MTC on the left thyroid lobe and lymphocytic thyroiditis with clusters of hyperplastic Hürthle cells. The tumour was confined to the thyroid, and the 20 lymph nodes that were analysed showed no evidence of metastasis. A genetic analysis of all of the *RET* hotspot exons revealed the Y791F variant. Three years after surgery, the calcitonin levels of this patient remain undetectable (<2 pg/ml); her carcinoembryonic antigen (CEA) level was 1.6 ng/ml, and cervical ultrasonography showed no sign of relapse. Additionally, her clinical and biochemical screening results were negative for phaeochromocytoma and hyperparathyroidism.

Screening of this patient's family revealed no history of endocrine tumours. Her 32-year-old sister also carries the *RET* Y791F variant and presented a thyroid ultrasound exhibiting thyroiditis and no nodules and biochemical examinations showing no evidence of MTC (calcitonin levels <1 pg/ml) or primary hyperparathyroidism. She was followed by another service and inadvertently underwent thyroidectomy. Pathological analysis found no evidence of MTC or C-cell hyperplasia. Her mother (59 years old) and her two sons (18 and 11 years old) did not harbour the *RET* Y791F variant and presented low calcitonin levels. Her father, who was probably the obligatory carrier, died in a car accident at the age of 46 years with no signs of the disease. He was from Germany. The lack of a family history of MTC in this family with three carriers argues that the *RET* Y791F variant is not an endocrine-neoplasia-susceptibility variant. Unfortunately, similar to this family, other asymptomatic relatives with the Y791F variant have also undergone unnecessary thyroidectomies (Supplementary References).

Recently, we have identified another patient with MTC harbouring the *RET* Y791F variant from the city of Curitiba, Brazil. The patient, a 35-year-old woman, was diagnosed with a thyroid nodule. The patient underwent a thyroidectomy, and the final histological analysis revealed MTC. An extended *RET* analysis (exons 1–12 and 14–21) was performed, and no other mutations were identified. The patient has a 9-year-old daughter exhibiting normal thyroid ultrasound results. The patient also has a German background.

### A new elderly phaeochromocytoma patient harbouring *RET* Y791F without MTC

A *RET* Y791F germline variant was observed in a patient being followed at Santa Casa Hospital in Sao Paulo, who was referred to our laboratory for genetic testing at the University of Sao Paulo. The remaining *RET* hotspot exons were sequenced, and no variant was identified. The patient was a 74-year-old man diagnosed with a left adrenal incidentaloma (9×8.3×6.5 cm). He had shown hypertension for the past 14 years, in addition to exhibiting chronic atrial fibrillation and subclinical autoimmune hyperthyroidism. His surgical pathology was diagnostic of phaeochromocytoma (8.5 cm and 220 g), and the results of his thyroid ultrasound examination were compatible with thyroiditis without nodules. His calcitonin and calcium/parathyroid hormone levels were normal. No family history of tumours was reported, and the sequencing results for the *TMEM127* gene showed no mutations. This case is another example of an elderly individual harbouring the *RET* Y791F variant without any increased risk for MTC.

### *RET* Y791F in human tumours

**Figure 3:**
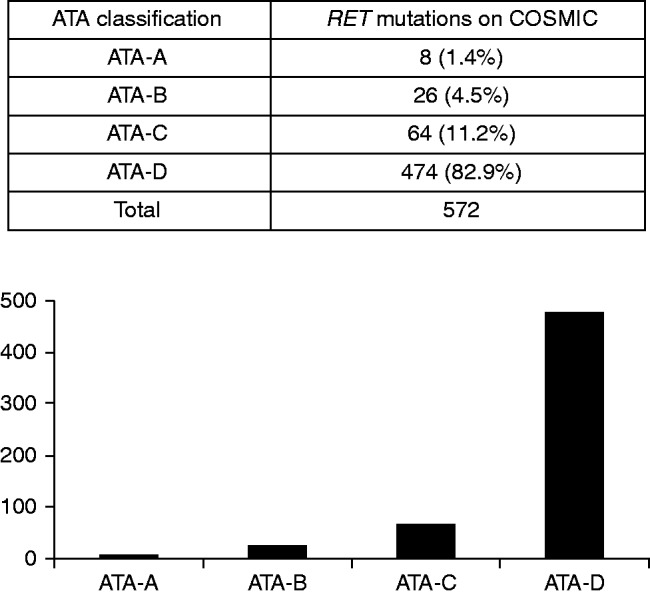
The number of *RET* mutations observed in the COSMIC cancer genetic and genomic dataset increases substantially according to the ATA mutation aggressiveness classification: 94.1% of the *RET* mutations found in cancers belong to the ATA C–D classification (strong mutations), while only 1.4% consist of weak ATA-A *RET* mutations. One of the 8 (12.5%) ATA-A variants is Y791F, corresponding to 0.17% of the mutations.

The number of *RET* ATA mutations observed in the COSMIC genetic and genomic dataset increased substantially according to the mutation's aggressiveness: 94.1% of the ATA *RET* mutations occurring in cancers belong to the ATA C–D classification (strong mutations), while only 1.4% are weak ATA-A *RET* mutations (Fig. 3 and Supplementary Tables 2 and 3, see section on supplementary data given at the end of this article). Among the ATA-A risk mutations, the most common variant is V804M, while the remaining variants are very rare and with an uncertain functional effect according to the ATA classification.

Among the entire COSMIC dataset of 18 163 tumour samples with available *RET* genotypes, only one (a lymphoid neoplasm, ID: COSM1159820) presented the *RET* Y791F variant (0.002% – no information available regarding somatic or germline status). Of the 1549 MTCs in the COSMIC dataset, 625 (40.3%) exhibited a *RET* mutation, none of which were Y791F, indicating that there is not a frequent association between this variant and MTC tumourigenesis (Supplementary Table 4, see section on supplementary data given at the end of this article).

In addition to the lack of evidence regarding a ‘driver’ property of *RET* Y791F in human tumours, two reports describing results indicative of a ‘passenger’ role for Y791F. The first case is an Y791F MTC patient in whom the most aggressive *RET* mutation, M918T, was identified somatically, which is most probably the cause of MTC (Fitze *et al*. 2002). The second case has been recently reported by Carlino *et al*. (2014) who described a patient with pancreatic carcinoma presenting the typical *KRAS* G12D mutation and the *RET* Y791F variant. There is no information as to whether the Y791F mutation was germline or somatic in this patient, but as *KRAS* G12D is a very well-described pancreatic cancer-driver mutation, the results strongly indicate a minor/non-pathological role for *RET* Y791F. Interestingly, the patient is Caucasian, was born in Australia and has a German surname (Carlino, electronic communication).

### *RET* Y791F in HSCR

*RET* Y791F was first described in a case of Hirschsprung's disease (HSCR) (Seri *et al*. 1997). HSCR is a genetically heterogeneous disorder, and inactivation (nonsense and frameshift) mutations in *RET* are its primary cause (Edery *et al*. 1994). In families harbouring activating *RET* mutations occurring primarily in extracellular domains (i.e. exon 10), such as C620R, it has been classically reported that HSCR develops in up to 7% of carriers. Since the initial report, other Y791F-carrier HSCR cases with no evidence of MTC have been reported, raising the question as to whether this variant is actually involved in tumourigenesis (Virtanen *et al*. 2013).

### *In silico* RET Y791F analyses

All four of the software programs used in this study predicted low or very low pathogenicity for the *RET* Y791F variant (data not shown). Although the software programs employ different algorithms to calculate potential pathogenicity, they all use evolutionary conservation, which indicated that an amino acid change at Tyrosine 791 is more likely to be tolerated, rather than being lethal or pathogenic. Our assessment of the RET protein structure indicate that Y791F localises to the N-lobe, in β 4, and is close to the ATP-binding site (Supplementary Fig. 3), which is a region that does not commonly contain strong *RET* mutations.

### *In vitro RET* Y791F assays

Plaza-Menacho *et al*. (2007) investigated the *RET* Y791F variant *in vitro* and reported ligand-independent activation. According to the results of this previous study, Y791F is an autophosphorylated monomeric activated receptor that leads to the constitutive activation of STAT3. However, the mechanism of STAT3 activation occurs via Src, JAK1 and JAK2, which differs from the classical *RET* C634R activation mechanism (Plaza-Menacho *et al*. 2007). In a later study, Hyndman *et al*. (2013) analysed *in vitro* *RET* variants that had been previously reported in HSCR cases and showed that Y791F behaved similarly to the WT protein in many respects, with similar RET/pERK/pSTAT3 phosphorylation, surface localisation, and RNA and protein stability being observed. Nevertheless, unlike to the RET WT protein and similarly to other HSCR variants, Y791F was associated with reduced levels of cell migration and no ability to promote colony formation (Hyndman *et al*. 2013). Although this last study did not include *bona fide RET*-activating mutations for comparison, the results indicate WT-like functional and molecular effects of Y791F, rather than Y791F acting as a gain-of-function mutation.

A third study by Cosci *et al*. (2011) combined *in silico* and *in vitro* assays to analyse known pathogenic mutations of *RET*, such as C634R and M918T and ‘rare medium/low-risk’ variants, including Y791F. Correlations between the results obtained using different approaches were observed primarily for the stronger variants, while the overall results and classification of the weaker variants remained unclear. The *in silico* classification of *RET* Y791F was deleterious, although focus and soft agar assays indicated Y791F to be a weak and probably non-pathogenic variant (Cosci *et al*. 2011). In contrast, the high proliferation rates associated with Y791F, C634R and M918T achieved statistical significance, but these results were inconclusive, as several probably non-pathogenic variants, such as V648I, also achieved statistical significance.

The three studies of *RET* cited above, together with many others in the literature, show how difficult and sometimes capricious it can be to characterise a weak variant using *in vitro* or *in silico* approaches, especially in genes related to genotypes with major medical implications, such as the recommendation for total thyroidectomy in the case of *RET*. To the best of our knowledge, there is no animal model of the *RET* Y791F variant.

### *RET* Y791F as a potential phenotypic modifying variant in cases with pathogenic *RET* mutations

The data that we were able to generate and review in the current study indicate a clear lack of pathogenicity of *RET* Y791F-only. However, the possible role of Y791F as a phenotype-modifying variant cannot be discarded. In fact, we have recently reported four MEN2A families carrying the classical *RET* C634Y mutation and Y791F *in cis* (Toledo 2010). The patients presented aggressive MTC (codon C634-like pattern) associated with an increased susceptibility to unusually large bilateral phaeochromocytomas. This finding is indicative of a modifier effect of Y791F when associated with classical disease-causing *RET* mutations (Toledo 2010). In the same study, we described *in vitro* analyses performed in Dr Lois Mulligan's laboratory (ON, Canada), demonstrating that the double C634Y/Y791F RET receptor is significantly more highly phosphorylated than either the WT receptor or the single C634Y and Y791F RET mutants (Toledo *et al*. 2010). Interestingly, an association between *RET* C634Y and Y791F has been recently observed in five additional Brazilian families (Cerutti & Maciel 2013, Valente *et al*. 2013). In addition to C634Y/Y791F, the presence of Y791F has been reported in patients harbouring other disease-associated *RET* mutations, such as C620F, V804L and M918T (Jindrichova *et al*. 2004). Although current knowledge shows no relevant clinical effect, the possible mechanism of such co-occurrence seems worth exploring in the future.

### Known *RET* missense variants identified through whole-exome/genome sequencing

Four variants classified as ATA-A mutations (S649L, V804M, R844Q and S891A) (Kloos *et al*. 2009) were rarely found in the whole-exome/genome sequences of the tumour-free individuals assessed in the current study. The V804M and S891A variants are also included in the *RET* list as MTC level 1 mutations (Brandi *et al*. 2001). As mentioned earlier, V804M is a well-described gatekeeper mutation associated with a mild, late-onset phenotype, which could explain its rare occurrence in the presumably healthy cohort (1/13 000 alleles in the ESP dataset, 0.00007). The frequencies of V804M and Y791F in the controls were very different and were compatible with a true, low-risk mutation and a rare non-pathogenic variant respectively (Fig. 2 and Supplementary Fig. 2, see section on supplementary data given at the end of this article). In addition, a novel change involving Valine 804 (c.2411C>T; p.V804A) was, to the best of our knowledge, identified for the first time in this study (Table 1, allelic frequency of 0.00001 in the SATX control cohorts). An *in silico* analysis using both SIFT and Polyphen2 indicated the V804M and V804A changes to be pathogenic.

In accordance with our results for S649L, seven individuals from the ESP dataset were also identified as harbouring this variant, which has been presumably associated with late-onset, non-aggressive MTC (Colombo-Benkmann *et al*. 2008).

*RET* V292M was also found in the SABE60+ cohort (1 out of 1218 of the sequenced alleles, 0.0008). This variant is classified as pathogenic in the ARUP MEN2 and dbSNP/NCBI databases, but it has not been included in the MTC guidelines (Eng *et al*. 1996, Kloos *et al*. 2009). Currently, limited evidence is available regarding the pathogenicity of this variant.

The previously reported *RET* Y791N variant was identified in two control individuals analysed via Sanger sequencing (2/2522, 0.008): a 76-year-old male and a 55-year-old female. There is no evidence of pathogenicity of this variant in the literature; it was initially reported in a case of HSCR and later in the sister of an MTC case harbouring *RET* R770Q (Ruiz-Ferrer *et al*. 2006, Frank-Raue *et al*. 2010).

### Novel *RET* missense variants identified via whole-exome/genome sequencing

Nine of the *RET* missense variants identified herein via massive parallel DNA sequencing have not been reported in the dbSNP/NCBI or ESP databanks and, to the best of our knowledge, are novel (Table 1). Eight of the variants were classified as damaging by Polyphen2 or SIFT; six were classified as damaging by both software programs; and one variant was classified as benign/tolerated by both software programs (Table 1). Despite the rarity of these variants and the *in silico* predictions, the fact that our population was composed of healthy adult/elderly individuals with no familial history of tumours indicates that these variants are probably benign polymorphisms or low-penetrance genetic changes. If new evidence appears in the future indicating pathogenicity in these variants, especially V804A, which occurs at a known *RET* hotspot, we will contact the carriers and offer genetic counselling, molecular testing and specific clinical screening.

## Conclusions of the current comprehensive assessment of *RET* Y791F

1. There is no robust evidence from either our data or the literature indicating that *RET* Y791F-only may cause familial MTC or MEN2 syndrome (reports addressing very small families should not be considered to be informative). *RET* Y791F, like other non-pathogenic variants of *RET*, can be found in sporadic MTC patients, but its presence alone does not indicate an increased MTC risk.
2. The frequencies of the *RET* Y791F variant detected in large cohorts of healthy individuals are characteristic of the behaviour of a rare benign polymorphism, rather than a medically actionable pathogenic mutation of the *RET* proto-oncogene.
  - Lessons from findings 1 and 2:
  - Asymptomatic germline *RET* Y791F-only carriers (with no other disease-causing mutations of *RET*) should not undergo preventive total thyroidectomy based on the genetics. Familial risk, clinical outcomes, and biochemical and imaging data are the most valuable resources for managing these cases.
  - There is no indication that genetic and clinical screening of family members of typical sporadic MTC cases harbouring *RET* Y791F-only is beneficial.
3. The patients with familial MTC/MEN2 who were initially reported to be *RET* Y791F-only carriers were also found to harbour other associated disease-causing *RET* variants (C634Y *in cis*), leading to the disease.
  - Lessons from finding 3:
  - Genetic testing for *RET* is only complete when sequencing of all of the hotspot mutations in exons 8 and 10–16 is successfully performed. That is, sequencing of the remaining exons should not cease when the first suspect variant is identified.
  - Sequencing or re-sequencing of the hotspot exons of the *RET* gene (and possibly the full gene depending on the phenotype) of familial MTC/MEN2 patients initially described as *RET* Y791F-only carriers can reveal medically actionable pathogenic mutations in the *RET* proto-oncogene.
  - Limitations of PCR, such as allelic drop-out (PCR failure of one allele) and/or preferential amplification (hypo-amplification of one allele), may be responsible for false-negative *RET* genetic results. Therefore, the use of a second set of primers (with different sequences than those used for the initial PCR and sequencing run) is recommended for the re-sequencing of questionable cases.
4. Phaeochromocytoma patients initially reported to be *RET* Y791F-only carriers can harbour variants of other genes, e.g. *VHL* or *SDHx*, that are most probably the cause of the disease.
  - Lesson from finding 4:
  - Established clinical predictors and algorithms should be used to sequence phaeochromocytoma-susceptibility genes in phaeochromocytoma cases that are initially reported as *RET* Y791F-only.
5. Biased genetic analysis of highly selected patients and a relatively small number of controls can result in misunderstanding and misclassification of the pathogenicity of rare variants in medically actionable genes.
  - Lesson from finding 5:
  - The analysis of large and informative datasets from tumour-free individuals constitutes an accessible and useful tool for improving the interpretation of a molecular diagnosis and aiding in genetic counselling and the clinical management and treatment of carriers.

## Final comments about the era of NGS

The scientific and medical community working on *RET*-related diseases needs to be aware of the implications related to the dramatic increase in the number of new variants of this gene being identified in the current next-generation sequencing era. In fact, nine new *RET* variants were identified in the present study. Moreover, it is imperative to use caution when interpreting genetic variants with limited information. At the same time, access to genetic data from large cohorts presents an opportunity to re-evaluate the clinical behaviour of disputed *RET* variants, such as Y791F.

## Websites

1. NHLBI ESP Exome Sequencing Project: http://evs.gs.washington.edu.
2. dbSNP/NCBI: http://www.ncbi.nlm.nih.gov/SNP.
3. Ensembl EBI/EMBL/WTSI: http://www.ensembl.org.
4. COSMIC: http://cancer.sanger.ac.uk/cancergenome/projects/cosmic.
5. ARUP RET database, Univ. Utah: http://arup.utah.edu/database/MEN2/MEN2_display.php.
6. SIFT: sift.jcvi.org.
7. PMUT: http://mmb2.pcb.ub.es:8080/PMut/.
8. POLYPHEN2: http://genetics.bwh.harvard.edu/pph2/.
9. Align-GVGD: http://agvgd.iarc.fr/.

## Supplementary data

This is linked to the online version of the paper at http://dx.doi.org/10.1530/ERC-14-0491.

Supplementary Data

## Figures and Tables

**Table 1:** The nine new variants of the *RET* proto-oncogene identified via whole-exome and whole-genome sequencing in the current study

**Casuistic**	**Nucleotide**	**Codon**	**RET protein**	**dbSNP or ESP**	**SIFT**	**PolyPhen2**	***n***	**Genotype frequency**
SABE60+	C1352T	451	T451M*	Not reported	D	B	1	0.0008
SATX	T1418A	473	L473Q	Not reported	D	D	3	0.0014
SATX	A1462T	488	T488S	Not reported	T	B	1	0.0004
SABE60+	C1573T	525	R525W	Not reported	D	D	1	0.0008
SABE60+	C1678T	560	P560S	Not reported	D	D	1	0.0008
SABE60+	G1799T	600	R600L	Not reported	D	B	1	0.0008
SATX	C2077T	693	R693C	Not reported	D	D	2	0.0001
SABE60+	C2225T	742	T742M	Not reported	D	D	1	0.0008
SABE60+	A2255G	752	Y752C	Not reported	T	D	1	0.0008
SATX	T2411C	804	V804A	Not reported	D	D	1	0.0005

Please see the discussion and interpretation of these findings in the main text. The T451M variant is not present in the control databases (dbSNP and ESP), but it was identified in two endometrium carcinomas (COSMIC project IDs: 1584958 and 918112). The other variants have not been reported in COSMIC. D, damaging; B, benign; T, tolerated; SABE60+, University of Sao Paulo; SATX, San Antonio, Texas.

## References

[bib1] Berndt I, Reuter M, Saller B, Frank-Raue K, Groth P, Grussendorf M, Raue F, Ritter MM, Höppner W (1998). A new hot spot for mutations in the *ret* protooncogene causing familial medullary thyroid carcinoma and multiple endocrine neoplasia type 2A. Journal of Clinical Endocrinology and Metabolism.

[bib2] Brandi ML, Gagel RF, Angeli A, Bilezikian JP, Beck-Peccoz P, Bordi C, Conte-Devolx B, Falchetti A, Gheri RG, Libroia A (2001). Guidelines for diagnosis and therapy of MEN type 1 and type 2. Journal of Clinical Endocrinology and Metabolism.

[bib3] Brauer VF, Scholz GH, Neumann S, Lohmann T, Paschke R, Koch CA (2004). *RET* germline mutation in codon 791 in a family representing 3 generations from age 5 to age 70 years: should thyroidectomy be performed?. Endocrine Practice.

[bib4] Carlino MS, Kwan V, Miller DK, Saunders CA, Yip D, Nagrial AM, Tomlinson J, Grimmond SM, Scolyer RA, Kefford RF (2014). New *RAS*-mutant pancreatic adenocarcinoma with combined BRAF and MEK inhibition for metastatic melanoma. Journal of Clinical Oncology.

[bib5] Carlomagno F, Guida T, Anaganti S, Vecchio G, Fusco A, Ryan AJ, Billaud M, Santoro M (2004). Disease associated mutations at valine 804 in the RET receptor tyrosine kinase confer resistance to selective kinase inhibitors. Oncogene.

[bib6] Cerutti JM, Maciel RM (2013). An unusual genotype–phenotype correlation in MEN 2 patients: should screening for RET double germline mutations be performed to avoid misleading diagnosis and treatment?. Clinical Endocrinology.

[bib7] Colombo-Benkmann M, Li Z, Riemann B, Hengst K, Herbst H, Keuser R, Gross U, Rondot S, Raue F, Senninger N (2008). Characterization of the *RET* protooncogene transmembrane domain mutation S649L associated with nonaggressive medullary thyroid carcinoma. European Journal of Endocrinology.

[bib8] Cosci B, Vivaldi A, Romei C, Gemignani F, Landi S, Ciampi R, Tacito A, Molinaro E, Agate L, Bottici V (2011). *In silico* and *in vitro* analysis of rare germline allelic variants of *RET* oncogene associated with medullary thyroid cancer. Endocrine-Related Cancer.

[bib9] Edery P, Lyonnet S, Mulligan LM, Pelet A, Dow E, Abel L, Holder S, Nihoul-Fékété C, Ponder BA, Munnich A (1994). Mutations of the *RET* proto-oncogene in Hirschsprung's disease. Nature.

[bib10] Eng C (2010). Mendelian genetics of rare – and not so rare – cancers. Annals of the New York Academy of Sciences.

[bib11] Eng C, Clayton D, Schuffenecker I, Lenoir G, Cote G, Gagel RF, van Amstel HK, Lips CJ, Nishisho I, Takai SI (1996). The relationship between specific RET proto-oncogene mutations and disease phenotype in multiple endocrine neoplasia type 2. International RET mutation consortium analysis. Journal of the American Medical Association.

[bib12] Erlic Z, Hoffmann MM, Sullivan M, Franke G, Peczkowska M, Harsch I, Schott M, Gabbert HE, Valimäki M, Preuss SF (2010). Pathogenicity of DNA variants and double mutations in multiple endocrine neoplasia type 2 and von Hippel-Lindau syndrome. Journal of Clinical Endocrinology and Metabolism.

[bib13] Feldman GL, Edmonds MW, Ainsworth PJ, Schuffenecker I, Lenoir GM, Saxe AW, Talpos GB, Roberson J, Petrucelli N, Jackson CE (2000). Variable expressivity of familial medullary thyroid carcinoma (FMTC) due to a *RET* V804M (GTG→ATG) mutation. Surgery.

[bib14] Fitze G, Schierz M, Bredow J, Saeger HD, Roesner D, Schackert HK (2002). Various penetrance of familial medullary thyroid carcinoma in patients with *RET* protooncogene codon 790/791 germline mutations. Annals of Surgery.

[bib15] Fitze G, Saeger HD, Roesner D, Schackert HK (2004). Management of multiple endocrine neoplasia syndrome type 2 families in association with rare germline mutations of the RET proto-oncogene. Klinische Pädiatrie.

[bib20] Frank-Raue K, Machens A, Scheuba C, Niederle B, Dralle H, Raue F, German MEN2 Study Group (2008). Difference in development of medullary thyroid carcinoma among carriers of RET mutations in codons 790 and 791. Clinical Endocrinology(Oxford).

[bib16] Frank-Raue K, Döhring J, Scheumann G, Rondot S, Lorenz A, Schulze E, Dralle H, Raue F, Leidig-Bruckner G (2010). New mutations in the RET protooncogene-L881V – associated with medullary thyroid carcinoma and -R770Q – in a patient with mixed medullar/follicular thyroid tumour. Experimental and Clinical Endocrinology & Diabetes.

[bib17] Freudenberg-Hua Y, Jan Freudenberg J, Vacic V, Abhyankar A, Emde AK, Ben-Avraham D, Barzilai N, Oschwald D, Christen E, Koppel J (2014). Disease variants in genomes of 44 centenarians. Molecular Genetics & Genomic Medicine.

[bib18] Gimm O, Niederle BE, Weber T, Bockhorn M, Ukkat J, Brauckhoff M, Thanh PN, Frilling A, Klar E, Niederle B (2002). RET proto-oncogene mutations affecting codon 790/791: a mild form of multiple endocrine neoplasia type 2A syndrome?. Surgery.

[bib19] Hyndman BD, Gujral TS, Krieger JR, Cockburn JG, Mulligan LM (2013). Multiple functional effects of RET kinase domain sequence variants in Hirschsprung disease. Human Mutation.

[bib41] Jindrichova S, Vcelak J, Vlcek P, Neradilova M, Nemec J, Bendlova B (2004). Screening of six risk exons of the RET proto-oncogene in families with medullary thyroid carcinoma in the Czech Republic. Journal of Endocrinology.

[bib21] Kloos RT, Eng C, Evans DB, Francis GL, Gagel RF, Gharib H, Moley JF, Pacini F, Ringel MD, Schlumberger M (2009). Medullary thyroid cancer: management guidelines of the American Thyroid Association. Thyroid.

[bib22] Krieger E, Joo K, Lee J, Lee J, Raman S, Thompson J, Tyka M, Baker D, Karplus K (2009). Improving physical realism, stereochemistry, and side-chain accuracy in homology modeling: four approaches that performed well in CASP8. Proteins.

[bib83] Li Y, Willer CJ, Ding J, Scheet P, Abecasis GR (2010). MaCH: using sequence and genotype data to estimate haplotypes and unobserved genotypes. Genetic Epidemiology.

[bib23] Margraf RL, Crockett DK, Krautscheid PM, Seamons R, Calderon FR, Wittwer CT, Mao R (2009). Multiple endocrine neoplasia type 2 *RET* protooncogene database: repository of MEN2-associated *RET* sequence variation and reference for genotype/phenotype correlations. Human Mutation.

[bib24] Pęczkowska M, Kowalska A, Sygut J, Waligórski D, Malinoc A, Janaszek-Sitkowska H, Prejbisz A, Januszewicz A, Neumann HP (2013). Testing new susceptibility genes in the cohort of apparently sporadic phaeochromocytoma/paraganglioma patients with clinical characteristics of hereditary syndromes. Clinical Endocrinology.

[bib25] Plaza-Menacho I, van der Sluis T, Hollema H, Gimm O, Buys CH, Magee AI, Isacke CM, Hofstra RM, Eggen BJ (2007). Ras/ERK1/2-mediated STAT3 Ser^727^ phosphorylation by familial medullary thyroid carcinoma-associated RET mutants induces full activation of STAT3 and is required for *c-fos* promoter activation, cell mitogenicity, and transformation. Journal of Biological Chemistry.

[bib26] Plaza-Menacho I, Barnouin K, Goodman K, Martínez-Torres RJ, Borg A, Murray-Rust J, Mouilleron S, Knowles P, McDonald NQ (2014). Oncogenic RET kinase domain mutations perturb the autophosphorylation trajectory by enhancing substrate presentation in trans. Molecular Cell.

[bib27] Ren J, Wen L, Gao X, Jin C, Xue Y, Yao X (2009). DOG 1.0: illustrator of protein domain structures. Cell Research.

[bib28] Rich TA, Feng L, Busaidy N, Cote GJ, Gagel RF, Hu M, Jimenez C, Lee JE, Perrier N, Sherman SI (2014). Prevalence by age and predictors of medullary thyroid cancer in patients with lower risk germline *RET* proto-oncogene mutations. Thyroid.

[bib29] Rückert F, Görgens H, Richter I, Krex D, Schackert G, Kuhlisch E, Fitze G, Saeger HD, Pilarsky C, Grützmann R (2011). *RET*-protooncogene variants in patients with sporadic neoplasms of the digestive tract and the central nervous system. International Journal of Colorectal Disease.

[bib30] Ruiz-Ferrer M, Fernández RM, Antiñolo G, López-Alonso M, Eng C, Borrego S (2006). A complex additive model of inheritance for Hirschsprung disease is supported by both RET mutations and predisposing *RET* haplotypes. Genetics in Medicine.

[bib31] Seri M, Yin L, Barone V, Bolino A, Celli I, Bocciardi R, Pasini B, Ceccherini I, Lerone M, Kristoffersson U (1997). Frequency of RET mutations in long- and short-segment Hirschsprung disease. Human Mutation.

[bib32] Tamanaha R, Camacho CP, Ikejiri ES, Maciel RM, Cerutti JM (2007). Y791F *RET* mutation and early onset of medullary thyroid carcinoma in a Brazilian kindred: evaluation of phenotype-modifying effect of germline variants. Clinical Endocrinology.

[bib34] Toledo SP, dos Santos MA, Toledo RA, Lourenço DM (2006). Impact of RET proto-oncogene analysis on the clinical management of multiple endocrine neoplasia type 2. Clinics (Sao Paulo).

[bib33] Toledo RA, Wagner SM, Coutinho FL, Lourenço DM, Azevedo JA, Longuini VC, Reis MT, Siqueira SA, Lucon AM, Tavares MR (2010). High penetrance of pheochromocytoma associated with the novel C634Y/Y791F double germline mutation in the *RET* protooncogene. Journal of Clinical Endocrinology and Metabolism.

[bib35] Valente FO, Dias da Silva MR, Camacho CP, Kunii IS, Bastos AU, da Fonseca CC, Simião HP, Tamanaha R, Maciel RM, Cerutti JM (2013). Comprehensive analysis of *RET* gene should be performed in patients with multiple endocrine neoplasia type 2 (MEN 2) syndrome and no apparent genotype–phenotype correlation: an appraisal of p.Y791F and p.C634Y RET mutations in five unrelated Brazilian families. Journal of Endocrinological Investigation.

[bib36] Vestergaard P, Vestergaard EM, Brockstedt H, Christiansen P (2007). Codon Y791F mutations in a large kindred: is prophylactic thyroidectomy always indicated?. World Journal of Surgery.

[bib37] Vierhapper H, Rondot S, Schulze E, Wagner L, Hanslik S, Niederle B, Bieglmayer C, Kaserer K, Baumgartner-Parzer S (2005). Primary hyperparathyroidism as the leading symptom in a patient with a Y791F RET mutation. Thyroid.

[bib38] Virtanen VB, Pukkala E, Kivisaari R, Salo PP, Koivusalo A, Arola J, Miettinen PJ, Rintala RJ, Perola M, Pakarinen MP (2013). Thyroid cancer and co-occurring *RET* mutations in Hirschsprung disease. Endocrine-Related Cancer.

[bib39] Weber F, Eng C (2005). Editorial: Germline variants within RET: clinical utility or scientific playtoy?. Journal of Clinical Endocrinology and Metabolism.

[bib40] Wells SA, Gosnell JE, Gagel RF, Moley J, Pfister D, Sosa JA, Skinner M, Krebs A, Vasselli J, Schlumberger M (2010). Vandetanib for the treatment of patients with locally advanced or metastatic hereditary medullary thyroid cancer. Journal of Clinical Oncology.

