# Supplementary material for: Comprehensive assessment of the disputed RET Y791F variant shows no association with medullary thyroid carcinoma susceptibility
Source: Endocr Relat Cancer. 2014 Nov 25;22(1):65–76. doi: 10.1530/ERC-14-0491 (PMC4289937; doi:10.1530/ERC-14-0491)
Supplement: Supplementary Data [file supp_22_1_65__index.html]

Comprehensive assessment of the disputed RET Y791F variant shows no association with medullary thyroid carcinoma susceptibility — MTC not related to RET Y791F — Supplementary Data 

# Comprehensive assessment of the disputed *RET* Y791F variant shows no association with medullary thyroid carcinoma susceptibility

## Supplementary Data

**Files in this Data Supplement:**

- Supplementary Figure 1 - (PDF 42 KB)
- Supplementary Figure 2 - (PDF 43 KB)
- Supplementary Figure 3 - (PDF 65 KB)
- Supplementary Information 1 - (PDF 133 KB)
- Supplementary Table 1 - (XLSX 35 KB)
- Supplementary Table 2 - (XLSX 51 KB)
- Supplementary Table 3 - (XLSX 37 KB)
- Supplementary Table 4 - (XLSX 106 KB)
