# Supplementary material for: Comprehensive assessment of the disputed RET Y791F variant shows no association with medullary thyroid carcinoma susceptibility
Source: Endocr Relat Cancer. 2014 Nov 25;22(1):65–76. doi: 10.1530/ERC-14-0491 (PMC4289937; doi:10.1530/ERC-14-0491)
Supplement: Supplementary Data [file supp_ERC-14-0491_Supplementary_figure_2.pdf]

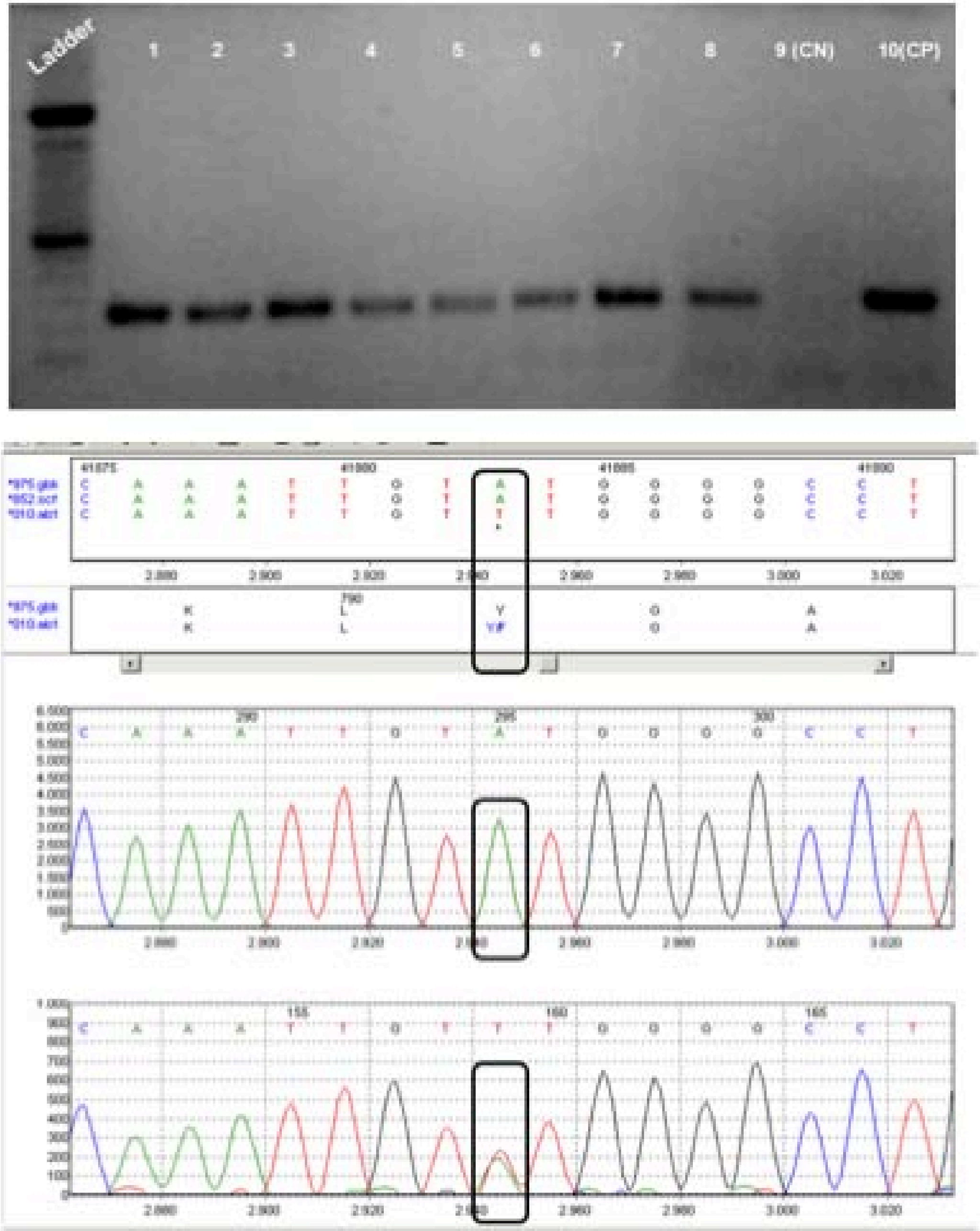
Ladder
1
2
3
4
5
6
7
8
9 (CN)
10(CP)
