# Supplementary material for: Comprehensive assessment of the disputed RET Y791F variant shows no association with medullary thyroid carcinoma susceptibility
Source: Endocr Relat Cancer. 2014 Nov 25;22(1):65–76. doi: 10.1530/ERC-14-0491 (PMC4289937; doi:10.1530/ERC-14-0491)
Supplement: Supplementary Data [file supp_ERC-14-0491_Supplementary_information_1.pdf]

SUPPLEMENTARY REFERENCES

Baumgartner-Parzer SM, Lang R, Wagner L, Heinze G, Niederle B, Kaserer K, Waldhäusl W & Vierhapper H 2005 Polymorphisms in exon 13 and intron 14 of the RET protooncogene: genetic modifiers of medullary thyroid carcinoma? J. Clin Endocrinol Metab 90 6232-6236.

Berndt I, Reuter M, Saller B, Frank-Raue K, Groth P, Grussendorf M, Raue F, Ritter MM & Höppner W 1998 A new hot spot for mutations in the ret protooncogene causing familial medullary thyroid carcinoma and multiple endocrine neoplasia type 2A. J Clin Endocrinol Metab 83 770-4.

Brandi ML, Gagel RF, Angeli A, Bilezikian JP, Beck-Peccoz P, Bordi C, Conte-Devolx B, Falchetti A, Gheri RG, Libroia A et al. 2001 Guidelines for diagnosis and therapy of MEN type 1 and type 2. J Clin Endocrinol Metab 86 5658-71.

Brauckhoff M, Gimm O, Hinze R, Ukkat J, Brauckhoff K & Dralle H 2002 Papillary thyroid carcinoma in patients with RET proto-oncogene germline mutation. Thyroid 12 557–561.

Brauer VF, Scholz GH, Neumann S, Lohmann T, Paschke R & Koch CA 2004 RET germline mutation in codon 791 in a family representing 3 generations from age 5 to age 70 years: should thyroidectomy be performed? Endocr Pract 10 5-9.

Cardot-Bauters C, Leteurtre E, Leclerc L, Vantyghem MC, Do Cao C, Wemeau JL, d'Herbomez M, Carnaille B, Barbu V, Pinson S et al. 2008 Does the RET variant G691S influence the features of sporadic medullary thyroid carcinoma? Clin Endocrinol 69 506-510.

Carlino MS, Kwan V, Miller DK, Saunders CA, Yip D, Nagrial AM, Tomlinson J, Grimmond SM, Scolyer RA, Kefford RF, *et al.* New RAS-Mutant Pancreatic Adenocarcinoma With Combined BRAF and MEK Inhibition for Metastatic Melanoma. J Clin Oncol. 2014 in press PMID: 24821886.

Carlomagno F, Guida T, Anaganti S, Vecchio G, Fusco A, Ryan AJ, Billaud M &Santoro M 2004Disease associated mutations at valine 804 in the RET receptor tyrosine kinase confer resistance to selective kinase inhibitors. Oncogene 23 6056-63.

Cebrian A, Lesueur F, Martin S, Leyland J, Ahmed S, Luccarini C, Smith PL, Luben R, Whittaker J, Pharoah PD et al. 2005 Polymorphisms in the initiators of RET (rearranged during transfection) signalling pathway and susceptibility to sporadic medullary thyroid carcinoma. J Clin Endocrinol Metab 90 6268-6274.

Cerutti JM & Maciel RM 2013 An unusual genotype-phenotype correlation in MEN 2 patients: should screening for RET double germline mutations be performed to avoid misleading diagnosis and treatment? Clin Endocrinol 79 591-2.

Colombo-Benkmann M, Brämswig J, Höppner W, Gellner R, Hengst K, Böcker W & Senninger N 2002 Surgical strategy in a kindred with a rare RET protooncogene mutation of variable penetrance with regard to multiple endocrine neoplasia. World J Surg 26 1286-90.

Colombo-Benkmann M, Li Z, Riemann B, Hengst K, Herbst H, Keuser R, Gross U, Rondot S, Raue F, Senninger N, et al. 2008 Characterization of the RET

protooncogene transmembrane domain mutation S649L associated with nonaggressive medullary thyroid carcinoma. Eur J Endocrinol 158 811-6.

Conzo G, Circelli L, Pasquali D, Sinisi A, Sabatino L, Accardo G, Renzullo A, Santini L, Salvatore F, Colantuoni V 2012 Lessons to be learned from the clinical management of a MEN 2A patient bearing a novel 634/640/700 mutation of the RET proto-oncogene. Clin Endocrinol 77 934-6.

Cosci B, Vivaldi A, Romei C, Gemignani F, Landi S, Ciampi R, Tacito A, Molinaro E, Agate L, Bottici V, et al. 2011 In silico and in vitro analysis of rare germline allelic variants of RET oncogene associated with medullary thyroid cancer. Endocr Relat Cancer 18 603-12

Donis-Keller H, Dou S, Chi D, Carlson KM, Toshima K, Lairmore TC, Howe JR, Moley JF, Goodfellow P & Wells SA Jr 1993 Mutations in the RET proto-oncogene are associated with MEN 2A and FMTC. Hum Mol Genet 2 851-856.

Edery P, Lyonnet S, Mulligan LM, Pelet A, Dow E, Abel L, Holder S, Nihoul-Fékété C, Ponder BA & Munnich A 1994 Mutations of the RET proto-oncogene in Hirschsprung's disease. Nature 367 378-80.

Elisei R, Romei C, Cosci B, Agate L, Bottici V, Molinaro E, Sculli M, Miccoli P, Basolo F, Grasso L et al. 2007 RET genetic screening in patients with medullary thyroid cancer and their relatives: experience with 807 individuals at one center. J Clin Endocrinol Metab 92 4725-4729.

Eng C 2010 Common alleles of predisposition in endocrine neoplasia. Curr Opin Genet Dev 20 251-6.

Eng C 2010 Mendelian genetics of rare--and not so rare--cancers. Ann N Y Acad Sci 1214 70-82.

Eng C, Clayton D, Schuffenecker I, Lenoir G, Cote G, Gagel RF, van Amstel HK, Lips CJ, Nishisho I, Takai SI, et al. 1996 The relationship between specific RET proto-oncogene mutations and disease phenotype in multiple endocrine neoplasia type 2. International RET mutation consortium analysis. JAMA 276 1575-9.

Erlic Z, Hoffmann MM, Sullivan M, Franke G, Peczkowska M, Harsch I, Schott M, Gabbert HE, Valimaki M, Preuss SF et al. 2010 Pathogenicity of DNA variants and double mutations in Multiple Endocrine Neoplasia type 2 and von Hippel-Lindau syndrome. J Clin Endocrinol Metab 95 308-313.

Feldman GL, Edmonds MW, Ainsworth PJ, Schuffenecker I, Lenoir GM, Saxe AW, Talpos GB, Roberson J, Petrucelli N & Jackson CE 2000 Variable expressivity of familial medullary thyroid carcinoma (FMTC) due to a RET V804M (GTG-->ATG) mutation. Surgery 128 93-8.

Fitze G, Paditz E, Schläfke M, Kuhlisch E, Roesner D, Schackert HK 2003 Association of germline mutations and polymorphisms of the RET proto-oncogene with idiopathic congenital central hypoventilation syndrome in 33 patients. J Med Genet 40:E10.

Fitze G, Saeger HD, Roesner D & Schackert HK 2004 Management of multiple endocrine neoplasia syndrome type 2 families in association with rare germline mutations of the RET proto-oncogene. Klin Padiatr 216 270-6.

Fitze G, Schierz M, Bredow J, Saeger HD, Roesner D & Schackert HK 2002 Various penetrance of familial medullary thyroid carcinoma in patients with RET protooncogene codon 790/791 germline mutations. Ann Surg 236 570-5.

Frank-Raue K, Buhr H, Dralle H, Klar E, Senninger N, Weber T, Rondot S, Höppner W & Raue F 2006 Long-term outcome in 46 gene carriers of hereditary medullary thyroid carcinoma after prophylactic thyroidectomy: impact of individual RET genotype. Eur J Endocrinol 155 229-36.

Frank-Raue K, Döhring J, Scheumann G, Rondot S, Lorenz A, Schulze E, Dralle H, Raue F & Leidig-Bruckner G 2010 New mutations in the RET protooncogene-L881V - associated with medullary thyroid carcinoma and -R770Q - in a patient with mixed medullar/follicular thyroid tumour. Exp Clin Endocrinol Diabetes 118 550-3.

Frank-Raue K, Heimbach C, Rondot S, Usadel KH, Meng W, Varma C, Fuchs-Hammoser R, Höppner W, Schulze E & Raue F 2003 Hereditary medullary thyroid carcinoma genotype-phenotype characterization. Dtsch Med Wochenschr 128 1998-2002.

Frank-Raue K, Machens A, Scheuba C, Niederle B, Dralle H, Raue F & German MEN2 Study Group 2008 Difference in development of medullary thyroid carcinoma among carriers of RET mutations in codons 790 and 791. Clin Endocrinol (Oxf) 69 259-63.

Frank-Raue K, Rondot S, Hoeppner W, Goretzki P, Raue F & Meng W 2005 Coincidence of multiple endocrine neoplasia types 1 and 2: mutations in the RET protooncogene and MEN1 tumor suppressor gene in a family presenting with recurrent primary hyperparathyroidism. J Clin Endocrinol Metab 90 5575.

Frank-Raue K, Rondot S, Schulze E & Raue F 2007 Change in the spectrum of RET mutations diagnosed between 1994 and 2006. Clin Lab 53 273-282.

Freudenberg-Hua Y, Jan Freudenberg J, Vladimir Vacic V, Avinash Abhyankar A, Anne-Katrin Emde AK, Danny Ben-Avraham D, Nir Barzilai N, Dayna Oschwald D, Erika Christen E, Jeremy Koppel J, *et al*., 2014 Disease variants in genomes of 44 centenarians. *Molecular Genetics & Genomic Medicine in press*

Gimm O, Niederle BE, Weber T, Bockhorn M, Ukkat J, Brauckhoff M, Thanh PN, Frilling A, Klar E, Niederle B et al. 2002 RET proto-oncogene mutations affecting codon 790/791: a mild form of multiple endocrine neoplasia type 2A syndrome? Surgery 132 952–959.

Gimm O, Ukkat J, Niederle BE, Weber T, Thanh PN, Brauckhoff M, Niederle B & Dralle H 2004 Timing and extent of surgery in patients with familial medullary thyroid carcinoma/multiple endocrine neoplasia 2A-related RET mutations not affecting codon 634. World J Surg 28 1312-6.

Hubner RA & Houlston RS 2006 Molecular advances in medullary thyroid cancer diagnosis. Clin Chim Acta 370 2-8.

Hyndman BD, Gujral TS, Krieger JR, Cockburn JG & Mulligan LM 2013 Multiple functional effects of RET kinase domain sequence variants in Hirschsprung disease. Hum Mutat 34 132-42.

Jindrichová S, Vcelák J, Vlcek P, Neradilová M, Nemec J, Bendlová B 2004 Screening of six risk exons of the RET proto-oncogene in families with medullary thyroid carcinoma in the Czech Republic. J Endocrinol 83:257-65.

Kloos RT, Eng C, Evans DB, Francis GL, Gagel RF, Gharib H, Moley JF, Pacini F, Ringel MD, et al. 2009 Medullary thyroid cancer: management guidelines of the American Thyroid Association. Thyroid 19 565-612.

Kouvaraki MA, Shapiro SE, Perrier ND, Cote GJ, Gagel RF, Hoff AO, Sherman SI, Lee JE & Evans DB 2005 RET proto-oncogene: a review and update of genotype-phenotype correlations in hereditary medullary thyroid cancer and associated endocrine tumors. Thyroid 15 531-544.

Krieger E, Joo K, Lee J, Lee J, Raman S, Thompson J, Tyka M, Baker D & Karplus K 2009 Improving physical realism, stereochemistry, and side-chain accuracy in homology modeling: Four approaches that performed well in CASP8. Proteins 77 Suppl 9 114-22.

Machens A & Dralle H 2007 Genotype-phenotype based surgical concept of hereditary medullary thyroid carcinoma. World J Surg 31 957-68.

Machens A, Brauckhoff M, Holzhausen HJ, Thanh PN, Lehnert H & Dralle H 2005 Codon-specific development of pheochromocytoma in multiple endocrine neoplasia type 2. J Clin Endocrinol Metab 90 3999-4003.

Machens A, Lorenz K, Dralle H 2014 Progression of medullary thyroid cancer in RET carriers of ATA class A and C mutations. J Clin Endocrinol Metab 99 286-92.

Machens A, Lorenz K, Sekulla C, Höppner W, Frank-Raue K, Raue F, Dralle H 2013 Molecular epidemiology of multiple endocrine neoplasia 2: implications for RET screening in the new millenium. Eur J Endocrinol. 168 307-14.

Machens A, Niccoli-Sire P, Hoegel J, Frank-Raue K, van Vroonhoven TJ, Roeher HD, Wahl RA, Lamesch P, Raue F, Conte-Devolx B et al. 2003 Early malignant progression of hereditary medullary thyroid cancer. N Engl J Med 349 1517-1525.

Margraf RL, Crockett DK, Krautscheid PM, Seamons R, Calderon FR, Wittwer CT, Mao R 2009 Multiple endocrine neoplasia type 2 RET protooncogene database: repository of MEN2-associated RET sequence variation and reference for genotype/phenotype correlations. Human Mutation 30 548-56.

Menacho IP, Koster R, Sloot AMV, Quax WJ, Osinga J, Sluis TV, Hollema H, Burzynski GM, Gimm O, Byus HCM et al. 2005 RET –Familial Medullary Thyroid Carcinoma Mutants Y791F and S891A Activate a Src/JAK/STAT3 Pathway, Independent of Glial Cell Line–Derived Neurotrophic Factor. Cancer Res 65 1729-1737.

Mulligan LM, Kwok JBJ, Healey CS, Elsdon MJ, Eng C, Gardner E, Love DR, Mole SE, Moore JK, Papi L et al. 1993 Germ-line mutations of the *RET* proto-oncogene in multiple endocrine neoplasia type 2. Nature 363 458-460.

Neumann HPH, Bausch B, McWhinney SR, Bender BU, Gimm O, Franke G, Schipper J, Klisch J, Altehoefer C, Zerres K et al. 2002 Germ-line mutations in nonsyndromic pheochromocytoma. N Engl J Med 346 1459-1466.

Pęczkowska M, Kowalska A, Sygut J, Waligórski D, Malinoc A, Janaszek-Sitkowska H, Prejbisz A, Januszewicz A, Neumann HP 2013 Testing new susceptibility genes

in the cohort of apparently sporadic phaeochromocytoma/paraganglioma patients with clinical characteristics of hereditary syndromes. Clin Endocrinol 79:817-23.

Plaza-Menacho I, Barnouin K, Goodman K, Martínez-Torres RJ, Borg A, Murray-Rust J, Mouilleron S, Knowles P & McDonald NQ 2014 Oncogenic RET kinase domain mutations perturb the autophosphorylation trajectory by enhancing substrate presentation in trans. Mol Cell 53 738-51.

Plaza-Menacho I, van der Sluis T, Hollema H, Gimm O, Buys CH, Magee AI, Isacke CM, Hofstra RM & Eggen BJ 2007 Ras/ERK1/2-mediated STAT3 Ser727 phosphorylation by familial medullary thyroid carcinoma-associated RET mutants induces full activation of STAT3 and is required for c-fos promoter activation, cell mitogenicity, and transformation. J Biol Chem 282 6415-24.

Qi XP, Ma JM, Du ZF, Ying RB, Fei J, Jin HY, Han JS, Wang JQ, Chen XL, Chen CY et al. 2011 RET germline mutations identified by exome sequencing in Chinese multiple endocrine neoplasia type 2A/familial medullary thyroid carcinoma family. PLoS One 6 e20353.

Quayle FJ, Fialkowski EA, Benveniste R & Moley JF 2007 Pheochromocytoma penetrance varies by RET mutation in MEN 2A. Surgery 142: 800-805.

Raue F & Frank-Raue K 2007 Multiple Endocrine Neoplasia Type 2: 2007 Update. Horm Res 68 101-104.

Raue F & Frank-Raue K 2009 Genotype-phenotype relationship in multiple endocrine neoplasia type 2. Implications for clinical management. HORMONES 8 23-28

Ren J, Wen L, Gao X, Jin C, Xue Y & Yao X 2009 DOG 1.0: illustrator of protein domain structures. Cell Res 19 271-3.

Rich TA, Feng L, Busaidy N, Cote GJ, Gagel RF, Hu M, Jimenez C, Lee JE, Perrier N, Sherman SI, et al. 2014 Prevalence by Age and Predictors of Medullary Thyroid Cancer in Patients with Lower Risk Germline RET Proto-Oncogene Mutations. Thyroid 24 1096-106.

Robledo M, Gil L, Pollan M, Cebrián A, Ruíz S, Azañedo M, Benitez J, Menárguez J & Rojas JM 2003 Polymorphisms G691S/S904S of RET as genetic modifiers of MEN 2A. Cancer Research 63 1814-1817.

Romei C, Mariotti S, Fugazzola L, Taccaliti A, Pacini F, Opocher G, Mian C, Castellano M, degli Uberti E, Ceccherini I et al. 2010 Multiple endocrine neoplasia type 2 syndromes (MEN 2): results from the ItaMEN network analysis on theprevalence of different genotypes and phenotypes. Eur J Endocrinol 163 963.

Rückert F, Görgens H, Richter I, Krex D, Schackert G, Kuhlisch E, Fitze G, Saeger HD, Pilarsky C, Grützmann R, et al. 2011 RET-protooncogene variants in patients with sporadic neoplasms of the digestive tract and the central nervous system. Int J Colorectal Dis 26 835-40.

Ruiz-Ferrer M, Fernández RM, Antiñolo G, López-Alonso M, Eng C &Borrego S 2006 A complex additive model of inheritance for Hirschsprung disease is supported by both RET mutations and predisposing RET haplotypes. Genet Med 8 704-10

Schuffenecker I, Virally-Monod M, Brohet R, Goldgar D, Conte-Devolx B, Leclerc L, Chabre O, Boneu A, Caron J, Houdent C et al. 1998 Risk and penetrance of

primary hyperparathyroidism in multiple endocrine neoplasia type 2A families with mutations at codon 634 of the RET proto-oncogene. Groupe d'Etude des Tumeurs a Calcitonine. J Clin Endocrinol Metab 83 487-491.

Seri M, Yin L, Barone V, Bolino A, Celli I, Bocciardi R, Pasini B, Ceccherini I, Lerone M, Kristoffersson U, et al. 1997 Frequency of RET mutations in long- and short-segment Hirschsprung disease. Hum Mutat 9 243-9.

Sromek M, Czetwertyńska M, Skasko E, Zielińska J, Czapczak D & Steffen J 2010 The frequency of selected polymorphic variants of the RET gene in patients with medullary thyroid carcinoma and in the general population of central Poland. Endocr Pathol 21 178-85.

Tamanaha R, Camacho CP, Ikejiri ES, Maciel RMB & Cerutti JM 2007 Y791F RET mutation and early onset of medullary thyroid carcinoma in a Brazilian kindred: evaluation of phenotype-modifying effect of germline variants. Clin Endocrinol 67 805-808.

Toledo RA, Wagner SM, Coutinho FL, Lourenço Jr DM, Azevedo JA, Longuini VC, Reis MTA, Siqueira SAC, Lucon AM, Tavares MR et al. 2010 High Penetrance of Pheochromocytoma associated with the novel C634Y/Y791F doublé germline mutation in the RET protooncogene. J Clin Endocrinol Metab 95 1318-1327.

Toledo SP, Lourenço DM Jr, Toledo RA 2013 A differential diagnosis of inherited endocrine tumors and their tumor counterparts. Clinics 68 1039-56

Vaclavikova E, Dvorakova S, Sykorova V, Bilek R, Dvorakova K, Vlcek P, Skaba R, Zelinka T & Bendlova B 2009 RET mutation Tyr791Phe: the genetic cause of different diseases derived from neural crest. Endocrinology 36 419–424.

Valente FO, Dias da Silva MR, Camacho CP, Kunii IS, Bastos AU, da Fonseca CC, Simião HP, Tamanaha R, Maciel RM & Cerutti JM 2013 Comprehensive analysis of RET gene should be performed in patients with multiple endocrine neoplasia type 2 (MEN 2) syndrome and no apparent genotype-phenotype correlation: an appraisal of p.Y791F and p.C634Y RET mutations in five unrelated Brazilian families. J Endocrinol Invest 36 975-81.

Vestergaard P, Vestergaard EM, Brockstedt H & Christiansen P 2007 Codon Y791F Mutations in a Large Kindred: Is Prophylactic Thyroidectomy Always Indicated? World Journal of Surgery 31 996-1001.

Vierhapper H, Bieglmayer C, Heinze G & Baumgartner-Parzer S 2004 Frequency of RET Proto-Oncogene Mutations in Patients with Normal and with Moderately Elevated Pentagastrin-Stimulated Serum Concentrations of Calcitonin. Thyroid 14 580- 583.

Vierhapper H, Rondot S, Schulze E, Wagner L, Hanslik S, Niederle B, Bieglmayer C, Kaserer K, Baumgartner-Parzer S 2005 Primary hyperparathyroidism as the leading symptom in a patient with a Y791F RET mutation. Thyroid 15 1303-8.

Virtanen VB, Pukkala E, Kivisaari R, Salo PP, Koivusalo A, Arola J, Miettinen PJ, Rintala RJ, Perola M, Pakarinen MP. 2013 Thyroid cancer and co-occurring RET mutations in Hirschsprung disease. Endocr Relat Cancer 20 595-602

Weber F & Eng C 2005 Editorial: Germline variants within RET: clinical utility or scientific playtoy? J Clin Endocrinol Metab 90 6334-6336.

Wells SA Jr, Gosnell JE, Gagel RF, Moley J, Pfister D, Sosa JA, Skinner M, Krebs A, Vasselli J & Schlumberger M 2010 Vandetanib for the treatment of patients with locally advanced or metastatic hereditary medullary thyroid cancer. J Clin Oncol 28 767-72.

Yip L, Cote GL, Shapiro SE, Ayers GD, Herzog CE, Sellin RV, Sherman SI, Gagel RF, Lee JE & Evans DB 2003 Multiple endocrine neoplasia type 2: evaluation of the genotype-phenotype relationship. Arch Surg 138 409-416.
